# Standardizing trait selection in Mendelian randomization studies concerning sarcopenia

**DOI:** 10.1002/jcsm.13463

**Published:** 2024-03-23

**Authors:** Mingchong Liu, Pengcheng Chen, Chensong Yang, Guixin Sun

**Affiliations:** ^1^ Department of Traumatic Surgery, Shanghai East Hospital, School of Medicine Tongji University Shanghai China; ^2^ China‐Japan Union Hospital of Jilin University Changchun China

Mendelian randomization (MR) is a method of using genetic variants as instrumental variables to assess the causal effect of exposures on disease outcomes. MR has gained increasing attention in the field of genetic epidemiology and has been applied to explore the causal associations in various diseases, including sarcopenia.^1^

Traditional observational studies are often limited in their ability to demonstrate causal relationships. Due to cost limitations and the fact that the development of sarcopenia is a long‐term process, randomized controlled trials are also difficult to conduct to confirm its causal factors.^2^ MR might be a useful method to estimate the potential causal factors of sarcopenia. Searching the term ‘Sarcopenia AND Mendelian Randomization’ in PubMed, we found more than 70 MR studies about sarcopenia. In these studies, many potential causal associations of sarcopenia had been reported, including lifestyle, related diseases and so on. Discovering these causal factors of sarcopenia may provide potential evidence for clinical investigation. However, there was still an issue to be noted when conducting MR studies for sarcopenia: selection of the sarcopenia trait.

MR was based on summary genome‐wide association study (GWAS) data. Usually, when we try to obtain the summary GWAS data, most of the diseases or their specific classifications in summary GWAS were defined clearly, for example, Crohn's disease in inflammatory bowel diseases.^3^ However, facing the sarcopenia trait, we may not easily find a defined GWAS for sarcopenia. The most significant reason was that the definition of sarcopenia was proposed in recent years, and the cohorts used for GWAS may have begun decades ago, so these cohorts may not include sarcopenia as a specific disease or a specific trait.

The most satisfactory method to address this issue might be to conduct an updated meta‐GWAS and to include sarcopenia as a specific and defined disease following the recognized guidelines. However, due to the multiple steps of diagnosing sarcopenia and the abnormal trait measurements, we may face significant challenges in integrating trait information for each participant.

Though GWAS for overall sarcopenia trait may be unavailable, researchers always try to explore the causal factors for sarcopenia by using sarcopenia‐associated traits.^1^, ^2^, ^4^ The selection of sarcopenia‐associated traits was various: Some studies may include only one trait,^5^ and some may choose up to six or more^2^; some may only select parts of sarcopenia trait, for example, only including muscle strength and muscle quantity traits while ignoring the physical performance traits.^6^ The sarcopenia‐associated trait selection needs to be standardized to better reflect the sarcopenia status comprehensively.

According to the EWGSOP2 consensus, the evaluation of sarcopenia includes three parts: muscle strength, muscle quantity or quality, and physical performance.^7^ Therefore, we believe the selection of sarcopenia‐associated traits should also meet the three parts. We summarized the usual GWAS information for sarcopenia‐associated traits in *Table* *1*. Following the definition of sarcopenia, we suggest that we should ensure that the selection of sarcopenia‐associated traits covers three characteristics of sarcopenia (muscle strength, muscle quantity or quality, and physical performance) when selecting sarcopenia‐associated traits in MR analysis. Including three parameters of sarcopenia measurement may better improve the accuracy and comprehensiveness of MR studies about sarcopenia.

**Table 1:** Usual GWAS information for sarcopenia‐associated traits

Parameters	Traits	Sample size	Sources
Muscle strength	Handgrip strength (EWGSOP)	256 523	PMID: 33510174
	Handgrip strength (FNIH)	256 523	PMID: 33510174
	Handgrip strength (right)	461 089	IEUID: ukb‐b‐10215
	Handgrip strength (left)	461 026	IEUID: ukb‐b‐7478
Muscle mass or muscle quality	Appendicular lean mass (BIA)	450 243	PMID: 33097823
	Whole‐body fat‐free mass	454 850	IEUID: ukb‐b‐13354
Physical performance	Usual walking pace	459 915	IEUID: ukb‐b‐4711
	Physical activity in the last 4 weeks	460 376	IEUID: ukb‐b‐15869
	Able to walk or cycle unaided for 10 min	69 537	IEUID: ukb‐b‐14149
	Moderate to vigorous physical activity levels	377 234	PMID: 29899525

## Conflict of interest

The authors declare that they have no competing interests.

## Funding

This study was supported by the Shanghai Pudong New Area Summit (Emergency Medicine and Critical Care) Construction Project (Grant No. PWYgf2021‐03).
